# Unravelling the cellular response to the SARS-COV-2 vaccine in inflammatory bowel disease patients on biologic drugs

**DOI:** 10.1038/s41598-023-50537-y

**Published:** 2023-12-27

**Authors:** Samuel J. Martínez-Domínguez, Sandra García-Mateo, Pilar Sainz-Arnal, Javier Martínez-García, Beatriz Gallego-Llera, María Jesús Lozano-Limones, Sandra Hidalgo, Carla J. Gargallo-Puyuelo, Marta Latre-Santos, Maria Mercedes Lourdes Nocito-Colon, Luis Martínez-Lostao, Engy Refaie, Maria Teresa Arroyo-Villarino, Marcela del Rio-Nechaevsky, Ariel Ramirez-Labrada, Julián Pardo, Fernando Gomollón, Pedro M. Baptista

**Affiliations:** 1 Digestive Diseases Department, University Hospital “Lozano Blesa”, Av. San Juan Bosco, nº 15. PC: 50009, Zaragoza, Spain; 2 Aragón Health Research Institute (IIS Aragón), Zaragoza, Spain; 3 University of Zaragoza, Zaragoza, Spain; 4 Immunology Department, University Hospital “Lozano Blesa”, Zaragoza, Spain; 5 Institute of Nanoscience and Material of Aragón (INMA), Zaragoza, Spain; 6 Scuola di Specializzazione in Chirurgia Generale, Università Degli Studi di Pavia Fondazione IRCCS Policlinico San Matteo, Pavia, Italy; 7 CIBEREnfermedadesRaras (CIBERER), Madrid, Spain; 8 Biomedical Engineering Department, Universidad Carlos III de Madrid, Madrid, Spain; 9 IIS-Fundación Jiménez Díaz, Madrid, Spain; 10 CIBER Enfermedades Infecciosas (CIBERINFEC), Madrid, Spain; 11 CIBER Enfermedades Hepáticas y Digestivas (CIBEREHD), Madrid, Spain; 12 Fundación ARAID, Zaragoza, Spain

**Keywords:** Gastroenterology, Inflammatory bowel disease

## Abstract

Suboptimal vaccine response is a significant concern in patients with Inflammatory Bowel Disease (IBD) receiving biologic drugs. This single-center observational study involved 754 patients with IBD. In Phase I (October 2020-April 2021), 754 IBD participants who had not previously received the SARS-CoV-2 vaccine, underwent blood extraction to assess the seroprevalence of SARS-CoV-2 infection and IBD-related factors. Phase II (May 2021-October 2021) included a subgroup of 52 IBD participants with confirmed previous SARS-CoV-2 infection, who were studied for humoral and cellular response to the SARS-CoV-2 vaccine. In Phase I, treatment with anti-TNF was associated with lower rates of seroconversion (aOR 0.25 95% CI [0.10–0.61]). In Phase II, a significant increase in post-vaccination IgG levels was observed regardless of biologic treatment. However, patients treated with anti-TNF exhibited significantly lower IgG levels compared to those without IBD therapy (5.32 ± 2.47 vs. 7.99 ± 2.59 U/ml, p = 0.042). Following vaccination, a lymphocyte, monocyte, and NK cell activation pattern was observed, with no significant differences between patients receiving biologic drugs and those without IBD treatment. Despite lower seroprevalence and humoral response to the SARS-CoV-2 vaccine in patients treated with anti-TNF, the cellular response to the vaccine did not differ significantly from that patients without IBD therapy.

## Introduction

Inflammatory Bowel Diseases (IBD), which comprise Ulcerative Colitis (UC), Crohn´s Disease (CD) and Indeterminate Colitis (IC), are chronic entities characterized by relapses of intestinal inflammation and periods of remission^1^. Immunosuppressive drugs (thiopurines, methotrexate), steroids and, more recently, biologic agents are frequently used to induce and/or maintain remission. Although the experience with biologic agents in patients with IBD is increasing, especially with anti-TNF drugs, multiple concerns have arisen about the risk of infections and responses to vaccines^2^.

Coronavirus disease-19 (COVID-19), caused by Severe Acute Respiratory Syndrome Coronavirus 2 (SARS-CoV-2), was first reported on December 2019 in Wuhan (China). Due to its rapid spread worldwide, the World Health Organization (WHO) declared a pandemic state on March 2020^3^.Since the beginning of the pandemic, seroprevalence studies have been carried out in the general population. However, it is of great interest to verify if these results are reproducible in high-risk populations such as IBD patients^4^.Whereas some studies did not report an increased risk of COVID-19 in patients with IBD^5–8^, other studies found an association between severe COVID-19 infection and IBD activity or some treatments^9–11^. Lower production of antibodies against SARS-CoV-2 has been found in patients receiving biologic agents^12^, which other studies could not confirm^13^.

Vaccine response in patients with IBD has been a topic of great interest for several decades. National and international scientific societies periodically publish guidelines on infection prevention and recommend vaccination at diagnosis of IBD, preferably, before immunosuppressant or biologic therapy. For vaccines based on attenuated viruses the main concern is adverse effects, so the immunosuppressant or biologic drug must be stopped several weeks before vaccination. It cannot be resumed until four weeks post-vaccination^14,15^. On the other hand, inactivated virus-based vaccines, non-replicating vector vaccines and modified RNA vaccines are safe in patients receiving immunosuppressant or biologic drugs and the main consideration is a possible lower vaccine response. Current guidelines recommend modifying the dosage of Influenza and Hepatitis B vaccines to optimize the vaccine response in patients under immunosuppressive or non-selective biologic treatment^14,15^.

Most studies assessed the humoral response to vaccines^16,17^; however, fewer studies evaluated the cellular response to vaccination in the IBD population under biologic therapy^18.^ Therefore, the lessons from the immune response to SARS-CoV-2 vaccines are an excellent opportunity to understand better the vaccine response in patients treated with biologic agents.

Hence, this study aimed to assess the effect of IBD-related features on the seroprevalence of SARS-CoV-2 infection and to assess the humoral and cellular response to the SARS-CoV-2 vaccine in patients with IBD.

## Results

### Phase 1: Seroprevalence of SARS-CoV-2 infection

#### Characteristics of the sample

**Figure 1:**
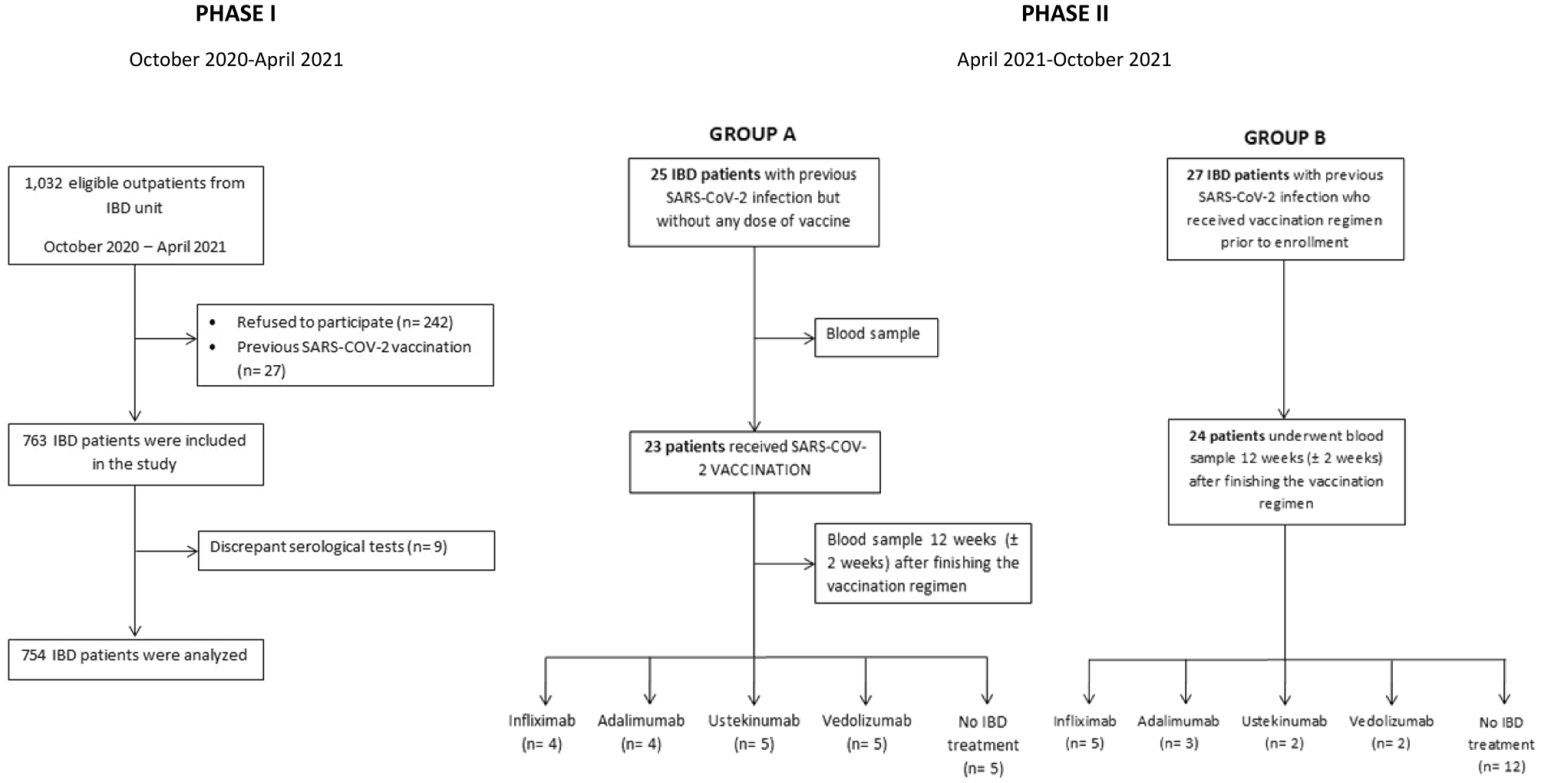
Flow chart of patient selection. *IBD* inflammatory bowel disease.

Of 1032 eligible outpatients from the IBD unit, 754 were analyzed (reasons for patient exclusion are shown in Fig. 1). 380 patients were men (50.4%), and the median age was 50 (18–86) years. 148 (19.6%) were active smokers, 350 (46.4%) were previous smokers, and 6 (0.8%) consumed illegal drugs. Main comorbidities were arterial hypertension (131 [17.7%]), hypercholesterolemia (140 [18.8%]), obesity (133 [17.6%]), chronic kidney disease (69 [9.4%]) and diabetes mellitus type 2 (50 [6.8%]).

The median age at diagnosis of IBD was 35 (1–77) years and 395 (52.3%) had UC, 350 (46.4%) CD and 9 (1.2%) IC. Most patients had extensive UC (155 [39.2%]) whereas 143 (36.2%) had left-sided UC and 97 (24.6%) proctitis. CD affected ileum-colon [L3] in 172 (49.1%) cases, ileum [L1] in 132 (37.7%), colon [L2] in 42 (12%) and upper gastrointestinal tract [L4] in 4 (1.1%) patients, presenting in most cases inflammatory pattern (193 (55.1%)). Most patients were in clinical remission with a partial Mayo score of 0 (0–6) and Harvey Bradshaw Index of 0 (0–12) points. Current treatment for IBD was: 5-ASA (255 [33.8%]), anti-TNF (161 [21.4%]), no treatment (106 [14.1%]), non-anti-TNF biologic drugs (82 [10.9%]), immunosuppressant (74 [9.8%]), immunosuppressant + anti-TNF (39 [5.2%]), steroids (10 [1.3%]), immunosuppressant + non-anti-TNF biologic drug (7 [0.9%]), tofacitinib (6 [0.8%]) and other (14 [1.8%]).

At enrolment, 67 (8.9%) had a history of confirmed SARS-CoV-2 infection, 37 (4.9%) had a history of probable SARS-CoV-2 infection and 649 (86.2%) had no history of known infection. Of all patients, most of them remained asymptomatic (660 [87.8%]) whereas 92 patients (12.2%) developed symptoms compatible with SARS-CoV-2 infection. Among patients with a history of confirmed/suspected SARS-CoV-2 infection, 12 (11.5%) were asymptomatic and the most common symptoms were fever (52 [50%]), asthenia (38 [36.5%]), anosmia/ageusia (27 [25.9%]) and cough (22 [21.2%]). Eight patients required hospitalization (1.1% of the total and 7.7% of patients with confirmed/suspected SARS-CoV-2 infection).

#### Seroprevalence and potential risk factors associated with seroconversion and severity of the infection

The first test measuring anti-inactivated native antigen IgG was negative in 686 (91%) cases, positive in 61 (8.1%) and indeterminate in 7 (0.9%). IgA test was negative in 721 (95.6%) cases, positive in 19 (2.5%) and indeterminate in 14 (1.9%). The second test assessing anti-spike IgG levels was positive in 53 (63.1%), negative in 22 (26.2%) and indeterminate in 9 (10.7%). After solving discrepancies between the different tests, 72 (9.5%) patients were considered seroconverted and 682 (90.5%) had a negative serology.

Among patients with confirmed/suspected infection, higher seroconversion rates were detected for hospitalized patients compared to outpatients (87.5% vs 36.5%, p = 0.007). However, no differences were observed between symptomatic and asymptomatic patients (33.3% vs 41.3%; p = 0.758).

Obesity was identified as a risk factor for seroconversion (univariable: 16.5% vs 8.1%, p = 0.005; multivariable: aOR 2.28 95%CI (1.32–3.94), p = 0.003) as well as an increased waist perimeter (univariable: 13.4% vs. 7.1%, p = 0.005; multivariable: aOR2.03 95%CI (1.22–3.40), p = 0.007). Treatment with anti-TNF was associated with lower rates of seroconversion (3.7% vs. 13.0%, p < 0.001), findings confirmed in multivariable analysis (Table 1). No differences in the proportion of confirmed and suspected SARS-CoV-2 infection were detected between patients treated with biologic drugs and those without IBD treatment (Supplementary Fig. 1).

**Table 1:** Association between IBD therapy, seroconversion and hospitalization.

Treatment	Seroconversion p-value*	Hospitalization p-value*
Anti-TNF	** < 0.001** **aOR0.25 95%CI (0.10–0.61), p = 0.002	0.316
Non-anti-TNF biologic drug	0.350	1.000
Immunosuppressant + anti-TNF	0.067	0.421
Immunosuppressant + non-anti-TNF biologic drug	0.602	1.000
Immunosuppressant	0.561	1.000
Steroids	0.627	0.201

Treatments were compared using patients receiving no treatment and/or5-ASA alone as the reference group.

*ASA* 5-aminosalicylic acid, *TNF* tumor necrosis factor.

*Univariable analysis.

**Multivariable analysis.

Significant values are in bold.

Other treatments showed no association with seroconversion rates (Table 1). No differences were observed by age, sex, legal or illegal drug consumption, presence of metabolic syndrome or other comorbidities. Age at diagnosis of IBD, type of IBD, presence of perianal disease or extraintestinal manifestations, surgery for IBD or activity were not associated with seroconversion.

No association between the risk of hospitalization and demographic variables, comorbidities, IBD-related factors or IBD therapy (Table 1) was found.

### Phase II: Immune response against SARS-CoV-2 vaccine

#### Characteristics of the sample

Of 754 IBD patients who participated in Phase I, 52 IBD participants with previously confirmed SARS-CoV-2 infection were recruited for Phase II. Group A was compounded by 25 patients not vaccinated against SARS-CoV-2 who underwent a blood extraction at enrolment and after vaccination (23 participants were finally vaccinated). Group B included 24 patients who underwent one blood extraction after SARS-CoV-2 vaccination. A representative sample of patients under each biologic drug and control patients without any IBD treatment were included in both groups (Fig. 1).

The mean age of participants included in Group B was significantly higher (51.8 ± 13.2 years vs. 42.0 ± 14.1 years; p = 0.015) because public health policies started vaccination in decreasing order of age. In both groups, more than half of the participants were female and over 75% had CD. The baseline characteristics of participants included in Phase 2 are detailed in Table 2.

**Table 2:** Characteristics of the participants included in Phase II.

Characteristics	Group A (n = 25)	Group B (n = 24)	p-value
Female, n (%)	15 (60.0)	13 (54.2)	0.680
Age (years), mean ± SD	42.0 ± 14.1	51.8 ± 13.2	**0.015**
Type of IBD, n (%)			
UC	4 (16.0)	6 (25.0)	0.496
CD	21 (84.0)	18 (75.0)	0.496
Extent of UC, n (%) (n = 10)			
Proctitis	0 (0.0)	1 (16.7)	1.000
Left-sided colitis	2 (50.0)	3 (50.0)	1.000
Extensive colitis	2 (50.0)	2 (33.3)	1.000
Extent of CD, n (%) (n = 39)			
Ileocolonic [L3]	8 (38.1)	10 (55.6)	0.276
Terminal ileum [L1]	10 (47.6)	5 (27.8)	0.204
Colonic [L2]	1 (4.8)	1 (5.6)	1.000
Ileocolonic [L3] + Upper disease [L4]	2 (9.5)	1 (5.6)	1.000
Terminal ileum [L1] + Upper disease [L4]	0 (0.0)	1 (5.6)	0.462
CD behaviour, n (%) (n = 39)			
Inflammatory	11 (52.4)	12 (66.7)	0.366
Stricturing	9 (42.9)	3 (16.7)	0.096
Penetrating	1 (4.8)	2 (11.1)	0.586
Stricturing + Penetrating	0 (0.0)	1 (5.6)	0.462

p-values in bold format indicate statistical significance (p < 0.05).

*CD* Crohn’s disease, *IBD* inflammatory bowel disease, *UC* ulcerative colitis.

*The Chi-square test or Fisher’s test was used for the qualitative variables, and quantitative variables t-student or Mann–Whitney U test, as appropriate.

#### Humoral response

**Figure 2:**
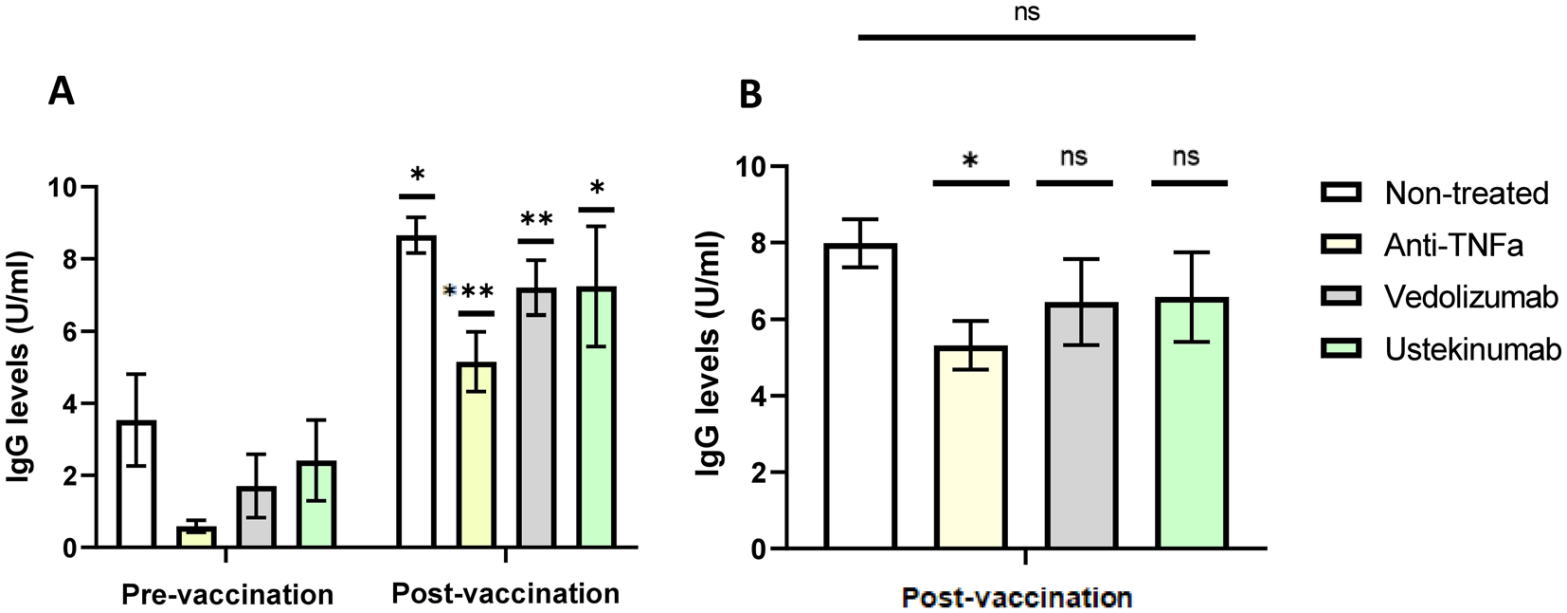
Humoral response to SARS-CoV-2 vaccine. (**A**) Comparison between pre and post-vaccination IgG levels in patients of Group A. (**B**) Comparison of post-vaccination IgG levels (vaccinated patients of Group A and B), detailed by treatment (patients with each biologic drug were compared with those without IBD treatment). ANOVA test was used. *p < 0.05 **p < 0.01 ***p < 0.001 ns: non-significant.

When IgG levels were compared before and after vaccination in Group A, a statistically significant increase was observed after immunisation, regardless of the biologic drug received (Supplementary Table 1). However, post-vaccination IgG levels in patients treated with anti-TNF were significantly lower compared with those without IBD treatment (Supplementary Table 2). No differences were observed for patients treated with ustekinumab and vedolizumab (Fig. 2).

Only 9 patients had a documented PCR (n = 3) or antigen test (n = 6) positive for SARS-CoV-2 in their medical records from vaccination to November 2023. We found no statistically significant differences in post-vaccination IgG levels when patients with/without a history of reinfection after vaccination were compared (7.51 ± 2.34 vs. 6.47 ± 2.97 U/ml, p = 0.333).

#### Cellular response

**Figure 3:**
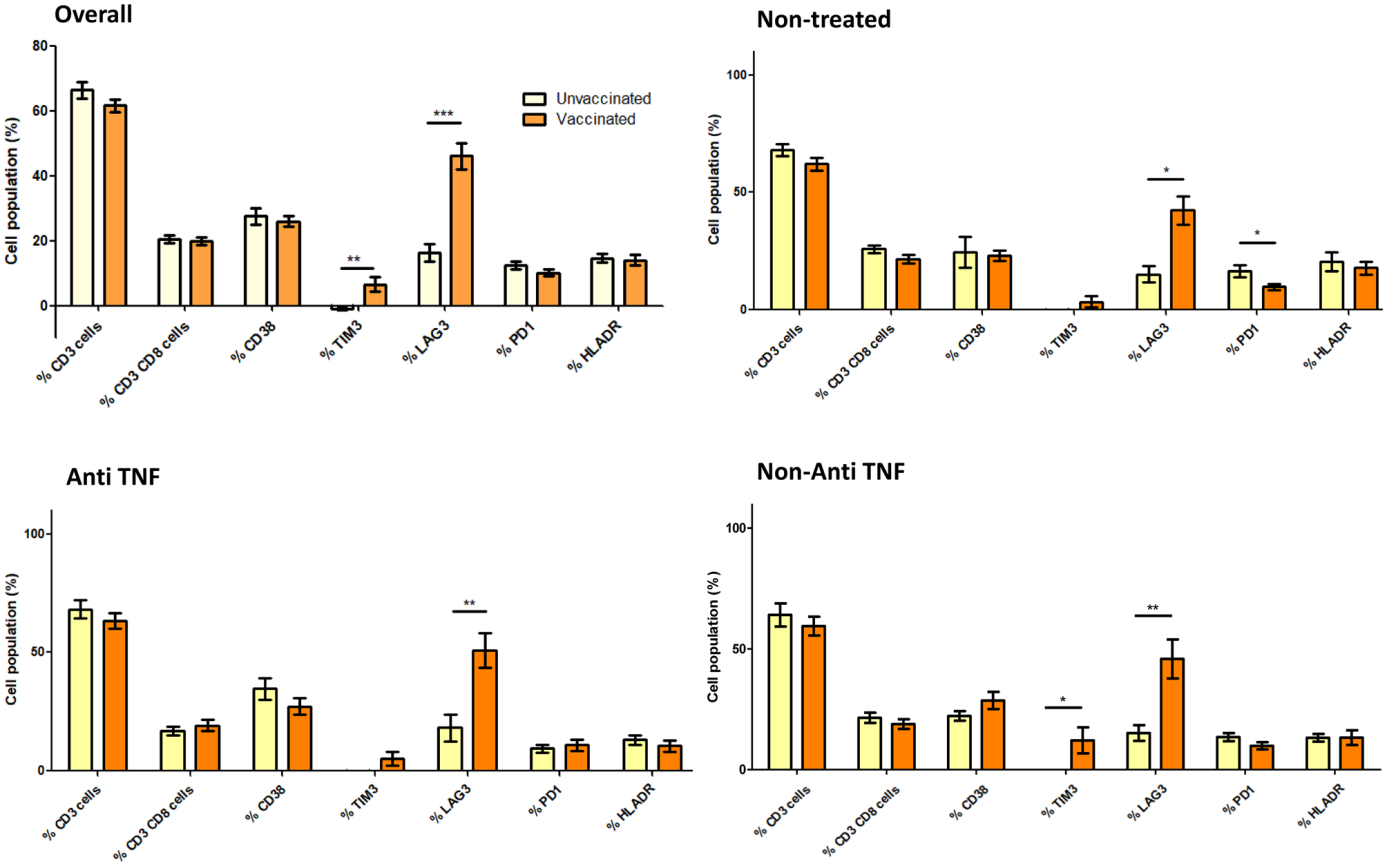
Cell populations in vaccinated and non-vaccinated patients: Lymphocytes. Unvaccinated: non-vaccinated patients of Group A. Vaccinated: vaccinated patients of Group A and Group B. *p < 0.05 **p < 0.01 ***p < 0.001.

**Figure 4:**
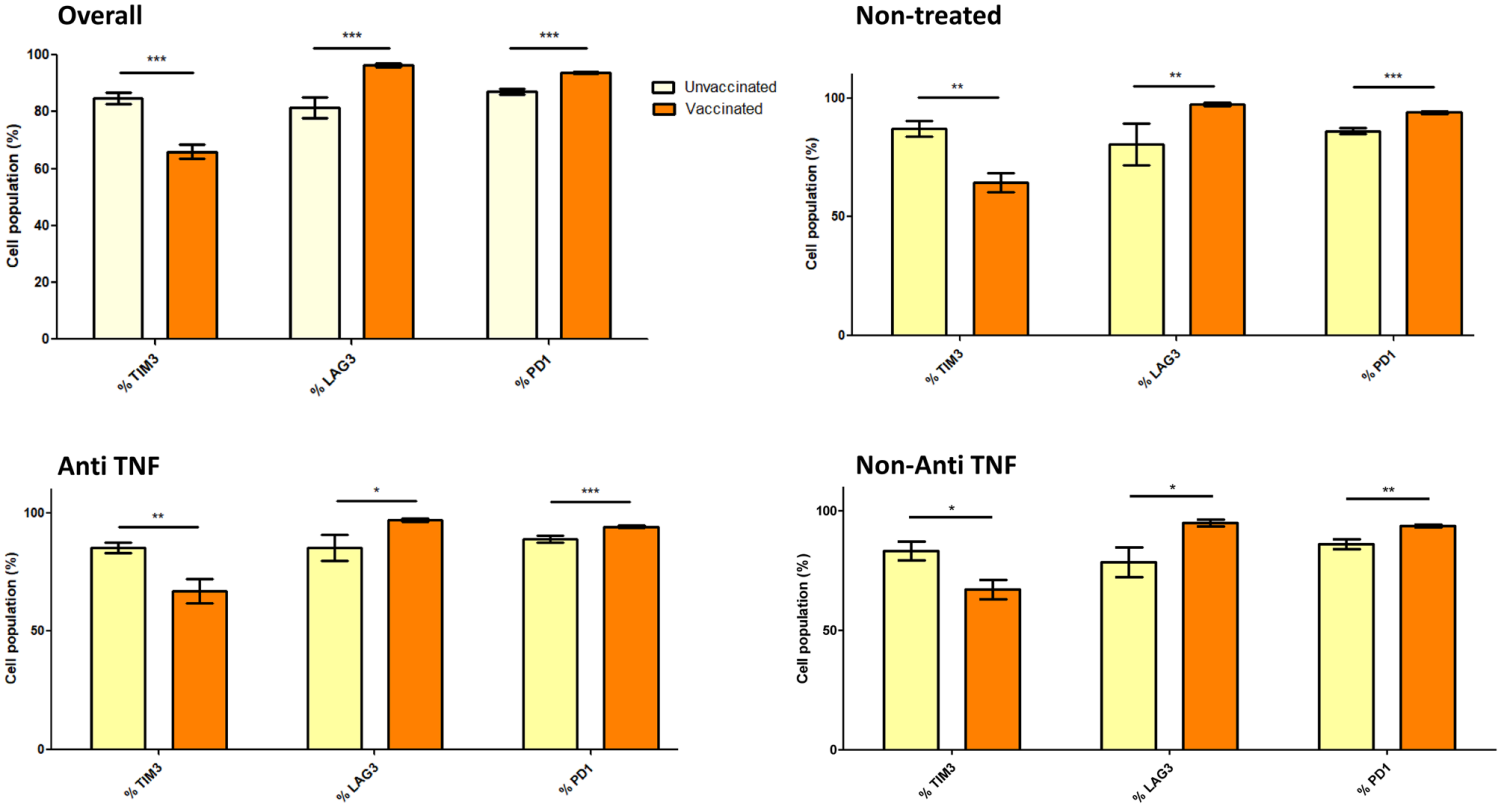
Cell populations in vaccinated and non-vaccinated patients: Monocytes. Unvaccinated: non-vaccinated patients of Group A. Vaccinated: vaccinated patients of Group A and Group B.*p < 0.05 **p < 0.01 ***p < 0.001.

**Figure 5:**
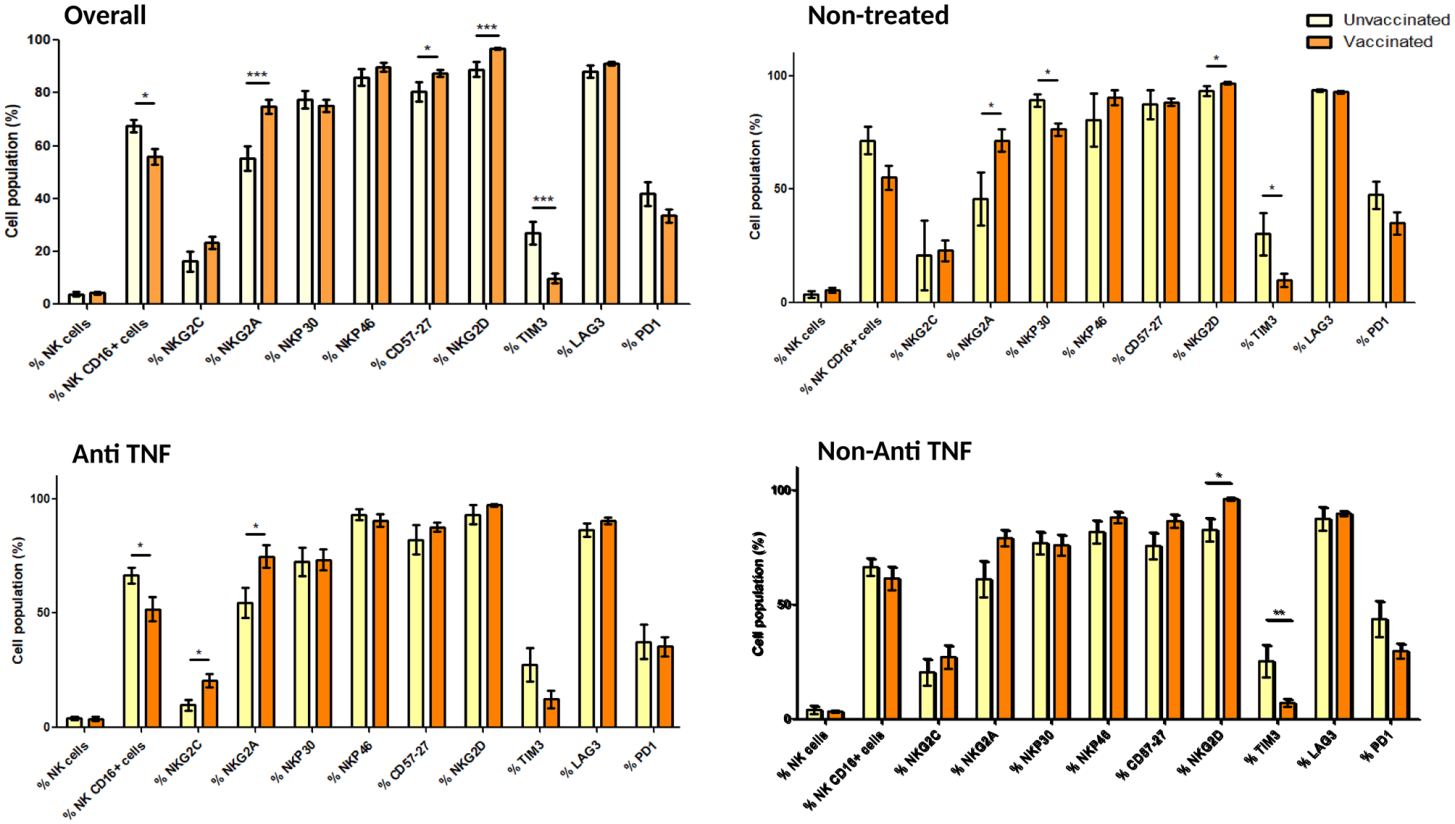
Cell populations in vaccinated and non-vaccinated patients: Natural Killer (NK). Unvaccinated: non-vaccinated patients of Group A. Vaccinated: vaccinated patients of Group A and Group B. *p < 0.05 **p < 0.01 ***p < 0.001.

After vaccination (vaccinated patients of Group A and B), the percentages of lymphocytes with LAG-3 and TIM-3 expression significantly increased compared with non-vaccinated patients (46.2 ± 27.6 vs. 16.4 ± 13.4%, p < 0.001 and 6.63 ± 14.3 vs. − 1.04 ± 1.09%, p = 0.010, respectively) (Fig. 3 and Supplementary Table 3). Regarding monocytes (Fig. 4 and Supplementary Table 4), response to vaccination significantly decreased the percentage of TIM-3 (66.0 ± 16.7 vs. 84.8 ± 9.4%, p < 0.001) and significantly increased the percentage of LAG-3 and PD-1 (96.4 ± 3.9 vs. 81.5 ± 18.3, p < 0.001 and 93.8 ± 2.3 vs. 87.2 ± 5.4%, p < 0.001, respectively). Regarding NK cells (Fig. 5 and Supplementary Table 5), activation after vaccination mainly increased the percentage of NKG2A (74.8 ± 17.7 vs. 55.3 ± 23.3%, p < 0.001) and NKG2D (96.6 ± 2.3 vs. 88.9 ± 13.9%, p < 0.001) and decreased the percentage of TIM-3 (9.8 ± 11.8 vs. 27.1 ± 21.5%, p < 0.001).

Opposite trends were observed between patients treated with anti-TNF and patients treated with vedolizumab. Detailed data of lymphocyte, monocyte and natural killer populations for vedolizumab and ustekinumab are showed in Supplementary Fig. 2. LAG-3 expression in the lymphocyte population was an excellent example of these differences. Patients treated with vedolizumab had a lower expression of this population than those receiving anti-TNF (Fig. 3 and Supplementary Table 3). Regarding monocyte populations, except for a decreasing tendency in the TIM-3 population with vedolizumab, no differences were observed between treated and untreated patients after vaccination (Fig. 4 and Supplementary Table 4). Finally, NK cell populations NKP30, CD57-27, TIM-3, and PD-1 showed different trends in patients treated with anti-TNF and vedolizumab (Fig. 5 and Supplementary Table 5).

When patients with CD and UC were compared, a higher increase of TIM-3 and LAG-3 lymphocytes, a more significant decrease of TIM-3 and PD-1 NK cells and a higher increase of NKG2A NK cells were observed (Supplementary Fig. 3). No conclusive differences were observed in the analysis stratified by gender (Supplementary Figs. 4–6).

## Discussion

Screening and prevention of infections in patients potentially receiving biologic drugs is an essential strategy, which is generally recommended before starting the treatment, due to a possible suboptimal vaccine response. SARS-CoV-2 infection is an excellent opportunity to learn about the immune response in patients treated with biologic drugs since it is a recently emerged disease with an available vaccine.

We found a significantly lower seroprevalence of SARS-CoV-2 infection and significantly lower post-vaccination IgG levels in patients treated with anti-TNF. Although conflicting data on the humoral response were published^13^ several studies reported an impaired humoral response to the SARS-CoV-2 vaccine in patients treated with anti-TNF drugs^12,19–22^. Thus, the hypothesis of possible suboptimal protection after SARS-CoV-2 vaccination in IBD patients treated with anti-TNF became more robust.

Nevertheless, the vaccine also induces a complex cellular response, which has been less studied in patients with IBD. Then, we studied several markers of activation or down-regulation of lymphocytes, monocytes and NK cells by flow cytometry. We chose specific markers based on two main reasons: 1. They were the main markers involved in the regulation of T and NK cell activation during viral infection; 2. A more detailed analysis using more markers was previously performed (Uranga et al.^35^) and these markers were the ones that showed more changes in these cell populations.

We identified an increased expression of LAG-3 and TIM-3 in lymphocytes of IBD patients after vaccination. LAG-3 is a transmembrane protein not expressed on naive T cells, but activation after antigen stimulation can promote its expression. The primary function of LAG-3 after binding to its ligand is the downregulation of immune response after activation. It reduces granzyme production in effector T cells and induces differentiation of regulatory T cells^23,24^. Similarly, TIM-3 also regulates effector T cell function^25^. The expression of co-inhibitory receptors usually results from cell activation to avoid an uncontrolled response^26^. When the different biologic drugs were compared, some differences in cell population trends were observed, although most did not reach statistical significance. HLA-DR, associated with T-cell activation, was decreased in patients treated with anti-TNF and vedolizumab. However, its role in regulating the antiviral immune response is still controversial^27^. For LAG-3, which is directly associated with a reduced immune response to viral infections, we observed a trend toward higher expression in patients treated with anti-TNF, in contrast to patients treated with vedolizumab.

Regarding monocytes, we found a significant increase in the proportion of PD-1 and LAG-3 after vaccination. PD-1 expression increases in monocytes as an activation marker and it has an inhibitory function to balance responses to clear pathogens and tumors and maintain tolerance. However, PD-1 expression showed a significant increase in anti-TNF-treated patients which could promote a decrease in the restoration of immune functions^28^. Lower TIM-3 expression in monocytes could promote immune stimulation through cytokine production and cytotoxicity of T cells^29^. TIM-3 expression tended to decrease in vedolizumab-treated patients. However, no other differences were found between other biologics and patients without IBD treatment^30^.

When we analyzed NK populations, an increased expression of NKG2A and NKG2D and a lower expression of TIM-3 were observed after vaccination. NKG2A are inhibitory receptors and NKG2D are activating receptors that recognise HLA-E and stress ligands (MIC and ULBP families) in infected target cells during antiviral responses^31,32^.Some studies reported that the spike 1 protein of SARS-CoV-2 induced overexpression of NKG2A and correlated with disease severity^33^. NKG2C transduces activation signals after recognizing non-classical HLA-E. In our study, patients treated with vedolizumab tended to have a higher expression of NKG2A, which could be associated with homeostatic control of the antiviral response as it is considered an immune checkpoint^34^. An opposite trend was observed in anti-TNF-treated patients, with lower expression of the NKG2C population that could promote lower expansion of memory NK cells providing a less efficient antiviral response.

Therefore, the activation patterns of the different cellular populations seemed interestingly similar when analyzed according to IBD therapy (Figs. 3, 4 and 5 and Supplementary Fig. 2). In summary, we observed similar trends in patients treated with vedolizumab and ustekinumab. However, they did not reach statistical significance occasionally, probably due to the smaller sample size of these subgroups. Despite the lower humoral response in patients who received anti-TNF drugs, our key finding was that these drugs did not significantly influence the cellular response compared with patients who did not receive IBD treatment.

Our findings in Phase II were plausible considering that most studies performed to date, and the Phase I of our study, found a similar severity and risk of infection in patients treated with anti-TNF compared with those without treatment^5–8^. It could be explained by the absence of relevant differences in cellular response, despite the lower serological response.

Finally, we acknowledge the following limitations of the study: 1. the small sample size of Phase II, despite post-vaccination patients were grouped, the pre-vaccination groups remained very small; 2. the small proportion of hospitalized patients could have influenced severity analysis in Phase I, and mortality could not be evaluated; 3. The analysis of cytokine production could have provided additional information on the functional activation of T/NK cells. However, the study main´s strength was the complete evaluation of the seroprevalence, potential IBD-related risk factors and humoral and cellular response to the vaccine. Other strengths were the internal control measuring levels of anti-TNF to ensure therapeutic adherence and the homogeneity of the sample and the diagnostic tests, because patients were recruited from the same healthcare area and samples were analyzed in the same equipment.

In conclusion, despite a lower seroprevalence and humoral response to the SARS-CoV-2 vaccine in patients treated with anti-TNF, cellular response to the vaccine did not significantly differ in patients treated with biologic drugs compared with patients who did not receive IBD therapy.

## Methods

### Study design and recruitment

This single-center observational study recruited consecutive outpatients treated in the IBD unit of the Lozano Blesa University Hospital of Zaragoza (Spain) between October 2020 and October 2021.

Patients older than 18 years with an established diagnosis of Crohn´s Disease (CD), Ulcerative Colitis (UC) or Indeterminate Colitis (IC) were included in the study, after signing informed consent.

**Figure 6:**
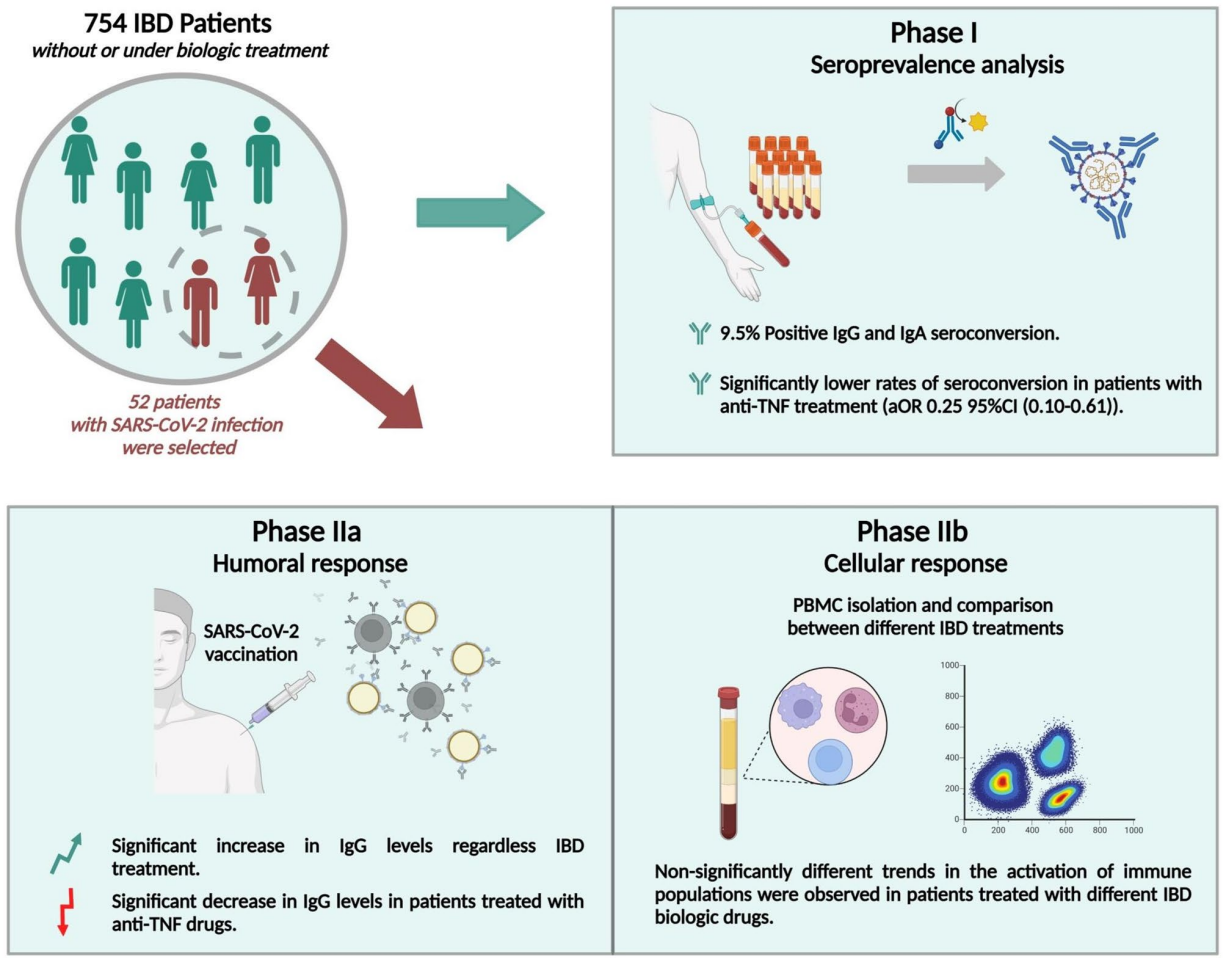
Study design: Phase I to assess SARS-CoV-2 seroprevalence and Phase II to assess humoral and cellular response to SARS-CoV-2 vaccine. *IBD* inflammatory bowel disease. *TNF* tumor necrosis factor.

To facilitate the monitoring and comprehension of the study, it was divided into 2 phases. Initially, Phase I to estimate the seroprevalence of SARS-CoV-2 infection in patients with IBD. Phase II to assess the humoral and cellular response to the SARS-CoV-2 vaccine in IBD patients under biologic therapy (Fig. 6).

#### Phase I: Seroprevalence

Between October 2020 and April 2021, 754 patients with IBD who had not previously been vaccinated against SARS-CoV-2 were included. All patients underwent a blood extraction saving several aliquots of each sample.

First, the ELISA method was used to measure IgG and IgA levels against inactivated native antigen of SARS-CoV-2 (Grifols®). Reference values recommended by the manufacturer were used for IgG and IgA (< 0.9 U/ml negative, 0.9–1.1 U/ml indeterminate, > 1.1 U/ml positive). In any case with a positive or indeterminate level of IgG and/or IgA, a confirmatory anti-spike protein IgG (Euroinmun®) test was performed, considering negative test in case of levels < 0.8 U/ml, positive if levels were > 1.1 U/ml and indeterminate between 0.8 and 1.1 U/ml.

Patients with a positive IgA and/or IgG test were considered seroconverted. Discrepant results between both IgG tests were interpreted as follows: in the case of IgG anti-spike positive the patient was considered to have seroconverted; cases with anti-inactivated native antigen IgG positive and anti-spike IgG negative or indeterminate were excluded.

#### Phase II: Humoral and cellular response to the SARS-CoV-2 vaccine

Between May 2021 and October 2021, the humoral and cellular response was determined in a subgroup of 52 patients with history of confirmed SARS-CoV-2 infection and IBD under biologic treatment (anti-TNF (infliximab or adalimumab), ustekinumab or vedolizumab) or without IBD treatment.

Participants were divided into two groups: Group A included patients with previous SARS-CoV-2 infection who had not received any dose of the SARS-CoV-2 vaccine at the beginning of Phase 2. Group B included patients with previous SARS-CoV-2 infection who had received the first dose of the SARS-CoV-2 vaccine at the beginning of Phase II.

Group A underwent a blood draw before receiving any dose of the SARS-CoV-2 vaccine and 12 weeks (± 2 weeks) after finishing theSARS-CoV-2 vaccination regimen. Group B underwent only one blood draw 12 weeks (± 2 weeks) after finishing the SARS-CoV-2 vaccination regimen. Due to the rapid implementation of the vaccination program in our area, we were unable to obtain pre-vaccination blood samples in group B because these patients had already received the vaccine at the time of recruitment.

Participants received, following national and local health authority guidelines, modified RNA based vaccines targeting the surface protein of SARS-CoV-2 (Moderna and Pfizer/BioNTech) or non-replicating vector vaccines which carry a SARS-CoV-2 antigen as transgene (Oxford-AstraZeneca).

The ELISA method described above was used again to measure IgG levels against inactivated native antigen (Grifols®) to analyse the humoral response.

For the analysis of the cellular response, peripheral blood was collected into sodium heparin tubes and treated with “Red Blood Cell Lysis Buffer” (Roche) for ten minutes at room temperature to remove the erythrocytes. Subsequently, the number of cells and viability were assessed and ten million of cells per patient were cryopreserved in a media composed of inactivated Fetal Bovine Serum (FBS) and Dimethyl Sulfoxide (DMSO). After collecting all samples from all participants, samples from each patient were thawed separately and incubated with DNase I (Roche) for 10 min at 37 °C to avoid aggregate formation. Finally, Peripheral Blood Mononuclear Cells (PBMCs) were counted and aliquoted for staining with fluorochloride-labeled antibodies and flow cytometry analysis (Gallios from Beckman Coulter). PBMCs were stained with specific antibodies using 3 different panels^35^. Panel 1 (T regulatory cells): CD3, CD8, CD45, CD38, CD8, HLADR, TIM3, PD1. Panel 2 (activating/inhibitory NK cell receptors): CD3, CD56, CD16, CD57, NKG2A, NKG2C, NKG2D, NKp30, NKp46. Panel 3 (activated/exhausted NK cells and Monocytes): CD3, CD56, CD16, CD14, TIM3, LAG3, PD1, HLA-DR.

To get a general view of T cell responses during infection we performed the analysis in the general T cell population. This was mainly based in previous studies in which significant changes were observed during SARS-CoV-2 infection correlating with disease severity when using similar selection strategies without requirement to analyze specific virus T cell responses. Indeed, although antigen specific T cell responses were analyzed in the previous studies, they did not correlate with disease severity^35^.

Detailed information on the antibodies used can be found in Supplementary Table 6. Flow cytometry gating strategy is detailed in Supplementary Fig. 7.

Additionally, a representative sample of patients under biologic treatment underwent drug-level analysis (Grifols®) to confirm treatment adherence.

### Variables

Medical records were reviewed to identify demographic variables, comorbidities, general blood tests (one month before or after the first consultation) and IBD-related features and its specific treatment. Localization or extension of IBD was defined using Montreal Classification for CD (ileal [L1], colonic [L2], ileocolonic [L3], isolated upper disease [L4]) or UC (proctitis, left-sided, pancolitis), respectively.

During the clinical history, IBD activity was assessed using validated questionnaires (partial Mayo score for UC and Harvey Bradshaw Index for CD). Clinical remission was considered in case of partial Mayo score ≤ 1 or Harvey-Bradshaw Index ≤ 4 points. Patients were classified by SARS-CoV-2 infection status according to the following definitions: confirmed SARS-CoV-2 infection in case of a positive SARS-CoV-2 PCR (Polymerase Chain Reaction) analysis taken by nasopharyngeal swab, and suspected SARS-CoV-2 infection if the participant had fever (≥ 38^○^C) with one or more respiratory tract symptoms without an alternative diagnosis in the absence of a PCR analysis.

### Statistical analysis and ethical declarations

Statistical analysis was performed using Jamovi version 2.3.21. Graphpad Prism was used to perform the graphs. Qualitative variables were presented as absolute and relative frequencies (n [%]). Normality was assessed using the Kolmogorov–Smirnov test where p > 0.05 means normality. Quantitative variables were presented as mean and standard deviation or median and range depending on normality. The Chi-square or Fisher exact test was used to study the association between qualitative variables, as appropriate. In the case of quantitative variables, Student´s t-Test, Analysis of Variance test (ANOVA), Mann–Whitney U Test or Wilcoxon test were used, as appropriate. A multivariable logistic regression analysis was also performed. Patients receiving no treatment and 5-Aminosalycilic Acid (5-ASA) were used as a reference group when an association between different treatments for IBD and seroconversion or severity was analyzed. A p-value < 0.05 was considered statistically significant.

There was no predefined sample size for Phase I, so all patients who attended our outpatient IBD unit during these months were invited to participate. For Phase II the sample size was limited, especially in Group A including patients recruited before vaccination, due to the start of the vaccination campaign and the low prevalence of confirmed SARS-CoV-2 infection (patients frequently had a history of suspected infection without microbiological confirmation).

This study was approved by the Ethics Committee of Clinical Research of Aragón (CEICA, Code PI20/478) and was carried out following the Declaration of Helsinki. All participants included in the study signed informed consent. Every data was confidential and subject to anonymization. Data are available on reasonable request from the authors. All authors had access to the study data and had reviewed and approved the final manuscript.

### Supplementary Information

Supplementary Information.

## Data Availability

Data available on reasonable request from the corresponding author (Samuel J. Martínez-Domínguez).
