# Supplementary material for: Unravelling the cellular response to the SARS-COV-2 vaccine in inflammatory bowel disease patients on biologic drugs
Source: Sci Rep. 2023 Dec 27;13:23061. doi: 10.1038/s41598-023-50537-y (PMC10754931; doi:10.1038/s41598-023-50537-y)
Supplement: Supplementary file 1 — Supplementary Information. [file 41598_2023_50537_MOESM1_ESM.docx]

**TITLE: UNRAVELLING THE CELLULAR RESPONSE TO THE SARS-COV-2 VACCINE IN INFLAMMATORY BOWEL DISEASE PATIENTS ON BIOLOGIC DRUGS**

Samuel J. Martínez-Domínguez^1,2,3,#,*^, Sandra García-Mateo^1,2,3,#^, Pilar Sainz-Arnal^2^, Javier Martínez-García^2,3^, Beatriz Gallego Llera^2^, María Jesús Lozano Limones^2^, Sandra Hidalgo^2,3^, Carla J. Gargallo-Puyuelo^1,2,3^, Marta Latre Santos^1^, Maria Mercedes Lourdes Nocito Colon^4^, Luis Martínez-Lostao^2,3,4,5^, Engy Refaie^6^, Maria Teresa Arroyo Villarino^1,2,3^, Marcela del Rio-Nechaevsky^7,8,9^, Ariel Ramirez-Labrada^2,3,10^, Julián Pardo^2,3,10^, Fernando Gomollón^1,2,3,11^, Pedro M. Baptista^2,8,11,12^

^1^University Hospital “Lozano Blesa”, Digestive Diseases Department, Zaragoza, Spain ^2^Aragón Health Research Institute (IIS Aragón) ^3^University of Zaragoza, Zaragoza, Spain ^4^University Hospital “Lozano Blesa”, Immunology Department, Zaragoza, Spain ^5^Institute of Nanoscience and Material of Aragón (INMA) ^6^Scuola di Specializzazione in Chirurgia Generale, Università degli Studi di Pavia Fondazione IRCCS Policlinico San Matteo, Pavia, Italy ^7^CIBER Enfermedades Raras (CIBERER), Madrid, Spain ^8^Biomedical Engineering Department Universidad Carlos III de Madrid, Madrid, Spain ^9^IIS-Fundación Jiménez Díaz, Madrid, Spain ^10^CIBER Enfermedades Infecciosas (CIBERINFEC), Madrid, Spain ^11^CIBER Enfermedades Hepáticas y Digestivas (CIBEREHD), Madrid, Spain^12^ Fundación ARAID

#Samuel J. Martínez-Domínguez and Sandra García-Mateo is dual first authorship due to equal contribution.

***Correspondence:** Samuel J. Martínez-Domínguez. Department of Digestive Diseases, University Hospital “Lozano Blesa”, Av. San Juan Bosco, nº 15. PC: 50009, Zaragoza, Spain. Telephone: +34976765700. Fax number: +34976765787. email: [samuelmartinez94@hotmail.com](mailto:samuelmartinez94@hotmail.com)

**SUPPLEMENTARY TABLES**

***Supplementary Table 1***. Humoral response to SARS-CoV-2 vaccine in Group A

| **GROUP A** | **PRE-VACCINATION IgG (U/ml)** | **POST-VACCINATION IgG (U/ml)** | **p-value*** |
| --- | --- | --- | --- |
| **Anti-TNF** | 0.66±0.57 | 5.16±2.35 | **<0.001** |
| **Ustekinumab** | 2.42±2.51 | 7.24±3.72 | **0.015** |
| **Vedolizumab** | 1.71±1.96 | 7.20±1.69 | **0.002** |
| **No IBD treatment** | 3.54±2.85 | 8.66±1.11 | **0.023** |

Data are expressed as mean ± SD (Standard Deviation). p values in bold format indicate statistical significance (p<0.05).

***Supplementary Table 2***. Humoral response to SARS-CoV-2 vaccine in Group A and B

| **POSTVACCINATED (GROUP A and GROUP B)** | **POST-VACCINATION IgG levels (U/ml)** | **p-value*** |
| --- | --- | --- |
| **Anti-TNF** | 5.32±2.47 | **0.042** |
| **Ustekinumab** | 6.58±3.30 | 0.633 |
| **Vedolizumab** | 6.45±2.97 | 0.598 |
| **No IBD treatment** | 7.99±2.59 |  |

Data are expressed as mean ± SD (Standard Deviation). *ANOVA test was used. Post-vaccination IgG levels of patients with each biologic drug were compared with those of patients without IBD treatment. p values in bold format indicate statistical significance (p<0.05).

***Supplementary Table 3.*** Lymphocyte populations in vaccinated and non-vaccinated patients

| **CELL POPULATION (PERCENTAGES)** | **VACCINATED (n= 47)** | **NON-VACCINATED (n= 23)** | **p-value** |
| --- | --- | --- | --- |
| ***Overall*** | | | |
| Lymphocytes (PBMC) | 61.7±12.6 | 66.5±12.3 | 0.125 |
| CD8 T lymphocytes | 20.0±8.2 | 20.5±6.5 | 0.785 |
| CD 38 | 26.1±11.9 | 27.7±12.8 | 0.601 |
| TIM3 | 6.63±14.3 | -1.04±1.09 | **0.010** |
| LAG3 | 46.2±27.6 | 16.4±13.4 | **<0.001** |
| PD1 | 10.2±6.9 | 12.5±5.9 | 0.162 |
| HLADR | 14.1±10.9 | 14.6±6.7 | 0.826 |
| ***No treatment*** | | | |
| Lymphocytes (PBMC) | 62.1±11.3 | 68.0±5.6 | 0.279 |
| CD8 T lymphocytes | 21.6±7.6 | 25.8±3.8 | 0.244 |
| CD 38 | 23.0±9.1 | 24.7±14.7 | 0.752 |
| TIM3 | 3.4±9.8 | -1.4±0.71 | 0.295 |
| LAG3 | 42.3±25.2 | 15.2±7.9 | **0.030** |
| PD1 | 9.8±5.5 | 16.5±5.8 | **0.028** |
| HLADR | 17.9±11.1 | 20.4±8.9 | 0.650 |
| ***Anti-TNF*** | | | |
| Lymphocytes (PBMC) | 63.3±12.5 | 68.2±12.2 | 0.341 |
| CD8 T lymphocytes | 19.2±9.7 | 16.9±5.3 | 0.489 |
| CD 38 | 27.2±13.4 | 34.6±14.6 | 0.202 |
| TIM3 | 5.1±11.3 | -0.87±1.25 | 0.111 |
| LAG3 | 50.9±28.8 | 18.2±18.4 | **0.004** |
| PD1 | 10.9±9.6 | 9.5±5.4 | 0.680 |
| HLADR | 10.5±9.2 | 13.1±5.9 | 0.440 |
| ***Vedolizumab*** | | | |
| Lymphocytes (PBMC) | 58.7±16.9 | 53.7±12.8 | 0.601 |
| CD8 T lymphocytes | 17.3±3.5 | 19.0±8.1 | 0.639 |
| CD 38 | 21.7±8.4 | 19.9±6.5 | 0.713 |
| TIM3 | 14.6±23.1 | -1.21±0.9 | 0.165 |
| LAG3 | 43.9±34.9 | 14.7±7.9 | 0.102 |
| PD1 | 10.1±4.3 | 13.8±4.4 | 0.194 |
| HLADR | 8.7±7.3 | 12.3±5.3 | 0.376 |
| ***Ustekinumab*** | | | |
| Lymphocytes (PBMC) | 59.9±13.7 | 74.4±9.4 | 0.065 |
| CD8 T lymphocytes | 20.1±9.6 | 23.9±4.9 | 0.426 |
| CD 38 | 33.8±14.4 | 24.5±5.8 | 0.202 |
| TIM3 | 10.3±19.0 | -0.9±1.4 | 0.224 |
| LAG3 | 47.2±28.4 | 15.6±13.3 | **0.042** |
| PD1 | 9.6±6.6 | 13.1±6.6 | 0.374 |
| HLADR | 16.7±13.1 | 14.1±5.3 | 0.684 |

PBMC: Peripheral Blood Mononuclear Cell. Data are expressed as mean ± SD (Standard Deviation).p values in bold format indicate statistical significance (p<0.05).

***Supplementary Table 4.*** Monocyte populations in vaccinated and non-vaccinated patients.

| **CELL POPULATION (PERCENTAGES)** | **VACCINATED (n= 47)** | **NON-VACCINATED (n= 23)** | **p-value** |
| --- | --- | --- | --- |
| ***Overall*** | | | |
| TIM3 | 66.0±16.7 | 84.8±9.4 | **<0.001** |
| LAG3 | 96.4±3.9 | 81.5±18.3 | **<0.001** |
| PD1 | 93.8±2.3 | 87.2±5.4 | **<0.001** |
| ***No treatment*** | | | |
| TIM3 | 64.4±16.0 | 87.1±7.1 | **0.007** |
| LAG3 | 97.2±3.2 | 80.6±19.6 | **0.002** |
| PD1 | 93.9±2.7 | 86.1±3.2 | **<0.001** |
| ***Anti-TNF*** | | | |
| TIM3 | 66.9±19.4 | 85.3±7.1 | **0.009** |
| LAG3 | 96.9±2.8 | 85.1±17.5 | **0.017** |
| PD1 | 94.1±2.2 | 88.9±4.8 | **0.001** |
| ***Vedolizumab*** | | | |
| TIM3 | 59.1±17.2 | 79.0±16.6 | 0.084 |
| LAG3 | 95.4±3.6 | 79.7±23.9 | 0.143 |
| PD1 | 93.2±2.9 | 83.8±6.3 | **0.010** |
| ***Ustekinumab*** | | | |
| TIM3 | 73.0±11.1 | 87.3±5.3 | **0.022** |
| LAG3 | 94.4±6.5 | 77.2±17.0 | **0.023** |
| PD1 | 93.9±1.1 | 88.1±6.6 | **0.031** |

Data are expressed as mean ± SD (Standard Deviation).p values in bold format indicate statistical significance (p<0.05).

***Supplementary Table 5.*** Natural Killer (NK) populations in vaccinated and non-vaccinated patients

| **CELL POPULATION (PERCENTAGES)** | **VACCINATED (n= 47)** | **NON-VACCINATED (n= 23)** | **p-value** |
| --- | --- | --- | --- |
| ***Overall*** | | | |
| NK cell (lymphocytes) | 4.3±3.6 | 3.9±4.0 | 0.656 |
| NK CD16+ (total NK) | 55.9±20.6 | 67.4±11.6 | **0.012** |
| NKG2C | 23.3±16.7 | 16.2±19.3 | 0.112 |
| NKG2A | 74.8±17.7 | 55.3±23.3 | **<0.001** |
| NKP30 | 75.1±14.9 | 77.5±16.9 | 0.539 |
| NKP46 | 89.7±11.3 | 85.9±16.3 | 0.258 |
| CD57-27 | 87.4±8.2 | 80.5±18.6 | **0.033** |
| NKG2D | 96.6±2.3 | 88.9±13.9 | **<0.001** |
| TIM3 | 9.8±11.8 | 27.1±21.5 | **<0.001** |
| LAG3 | 91.0±4.5 | 88.1±11.9 | 0.146 |
| PD1 | 33.5±16.9 | 41.9±22.3 | 0.079 |
| ***No treatment*** | | | |
| NK cell (lymphocytes) | 5.7±4.4 | 3.7±3.2 | 0.382 |
| NK CD16+ (total NK) | 55.2±21.9 | 71.4±13.5 | 0.135 |
| NKG2C | 22.9±18.7 | 20.9±34.5 | 0.863 |
| NKG2A | 71.4±20.1 | 45.8±26.1 | **0.029** |
| NKP30 | 76.3±10.9 | 89.2±6.2 | **0.022** |
| NKP46 | 90.3±13.5 | 80.4±26.1 | 0.258 |
| CD57-27 | 88.2±6.4 | 87.3±14.6 | 0.826 |
| NKG2D | 96.7±2.4 | 93.3±4.7 | **0.039** |
| TIM3 | 10.0±12.1 | 30.4±20.8 | **0.011** |
| LAG3 | 92.8±2.2 | 93.4±1.3 | 0.526 |
| PD1 | 35.0±20.5 | 47.4±13.7 | 0.222 |
| ***Anti-TNF*** | | | |
| NK cell (lymphocytes) | 3.8±3.6 | 3.9±2.3 | 0.914 |
| NK CD16+ (total NK) | 51.7±20.4 | 66.5±10.9 | **0.048** |
| NKG2C | 20.4±11.4 | 9.8±7.4 | **0.017** |
| NKG2A | 74.8±18.8 | 54.5±20.8 | **0.018** |
| NKP30 | 73.2±17.5 | 72.5±19.9 | 0.929 |
| NKP46 | 90.5±10.8 | 92.9±7.6 | 0.541 |
| CD57-27 | 87.5±7.6 | 82.1±20.6 | 0.359 |
| NKG2D | 97.1±1.8 | 92.9±13.2 | 0.239 |
| TIM3 | 12.3±14.9 | 27.4±22.9 | 0.057 |
| LAG3 | 90.4±6.1 | 86.3±9.3 | 0.197 |
| PD1 | 35.5±16.3 | 37.5±24.0 | 0.810 |
| ***Vedolizumab*** | | | |
| NK cell (lymphocytes) | 2.7±1.6 | 6.3±7.6 | 0.280 |
| NK CD16+ (total NK) | 61.8±22.7 | 68.8±13.9 | 0.560 |
| NKG2C | 24.2±14.6 | 15.2±13.6 | 0.321 |
| NKG2A | 80.3±12.7 | 56.9±23.8 | 0.067 |
| NKP30 | 77.7±17.9 | 75.4±19.0 | 0.841 |
| NKP46 | 85.1±11.7 | 85.9±14.7 | 0.919 |
| CD57-27 | 90.3±8.9 | 86.4±13.3 | 0.580 |
| NKG2D | 96.5±2.1 | 83.9±19.6 | 0.146 |
| TIM3 | 4.5±3.3 | 27.1±25.7 | 0.059 |
| LAG3 | 89.3±4.8 | 83.1±22.9 | 0.527 |
| PD1 | 29.4±14.8 | 37.8±29.1 | 0.549 |
| ***Ustekinumab*** | | | |
| NK cell (lymphocytes) | 3.5±1.8 | 1.4±1.3 | 0.051 |
| NK CD16+ (total NK) | 60.9±18.1 | 63.6±10.9 | 0.769 |
| NKG2C | 28.9±22.9 | 25.5±22.6 | 0.792 |
| NKG2A | 77.8±14.6 | 65.0±28.5 | 0.299 |
| NKP30 | 74.4±17.6 | 78.1±13.7 | 0.696 |
| NKP46 | 90.1±7.7 | 77.2±17.5 | 0.091 |
| CD57-27 | 83.4±11.9 | 64.6±17.1 | **0.038** |
| NKG2D | 95.5±2.9 | 81.2±14.1 | **0.016** |
| TIM3 | 8.8±7.9 | 23.2±21.6 | 0.108 |
| LAG3 | 89.8±3.9 | 91.6±5.3 | 0.491 |
| PD1 | 29.7±11.3 | 49.3±21.8 | 0.054 |

Data are expressed as mean ± SD (Standard Deviation).p values in bold format indicate statistical significance (p<0.05).

***Supplementary Table 6***. Detailed information on the antibodies used in Phase II

| **Antibody** | **Company** | **Reference** |
| --- | --- | --- |
| **CD14-VioBlue** | Miltenyi Biotec | 130-110-524 |
| **CD16-FITC** | Miltenyi Biotec | 130-113-392 |
| **CD3-VioGreen** | Miltenyi Biotec | 130-113-134 |
| **CD38-FITC** | Miltenyi Biotec | 130-113-426 |
| **CD56-PerCP Vio700** | Miltenyi Biotec | 130-100-681 |
| **CD57 APC-Vio770** | Miltenyi Biotec | 130-111-813 |
| **CD8-PerCP Vio700** | Miltenyi Biotec | 130-110-682 |
| **HLADR-APC-Vio770** | Miltenyi Biotec | 130-111-792 |
| **LAG3-APC** | Miltenyi Biotec | 130-119-567 |
| **NKG2A-PE-Vio615** | Miltenyi Biotec | 130-120-035 |
| **NKG2C-PE** | Miltenyi Biotec | 130-103-635 |
| **NKP46-APC** | Miltenyi Biotec | 130-092-609 |
| **30 TIM3-PE Vio770** | Miltenyi Biotec | 130-121-334 |
| **NKp30-PE/Cy** | Biolegend | 325214 |
| **PD1-Alexa Fluor700** | Biolegend | 329952 |
| **CD45-Brilliant Violet 421** | Biolegend | 304032 |
| **NKG2D-Brilliant Violet 421** | BD Biosciences | 743558 |

APC: Allophycocyanin. Cy: Cyanine. FITC: Fluorescein Isothiocyanate. PE: Phycoerythrin. PerCP: Peridinin-Chlorophyll-Protein.

**SUPPLEMENTARY FIGURES**


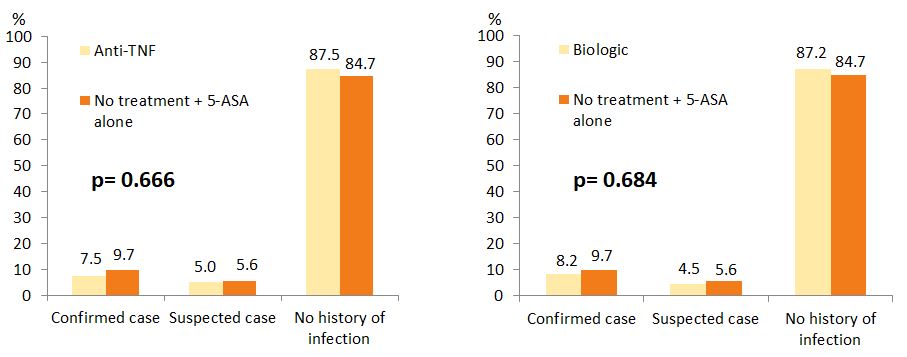


***Supplementary Figure 1***. Proportion of confirmed and suspected cases in patients treated with anti-TNF. Data are presented as percentages (%). The Chi-square test or Fisher´s test was used for the qualitative variables. ASA: 5-Aminosalicylic Acid. TNF: Tumor Necrosis Factor.

***
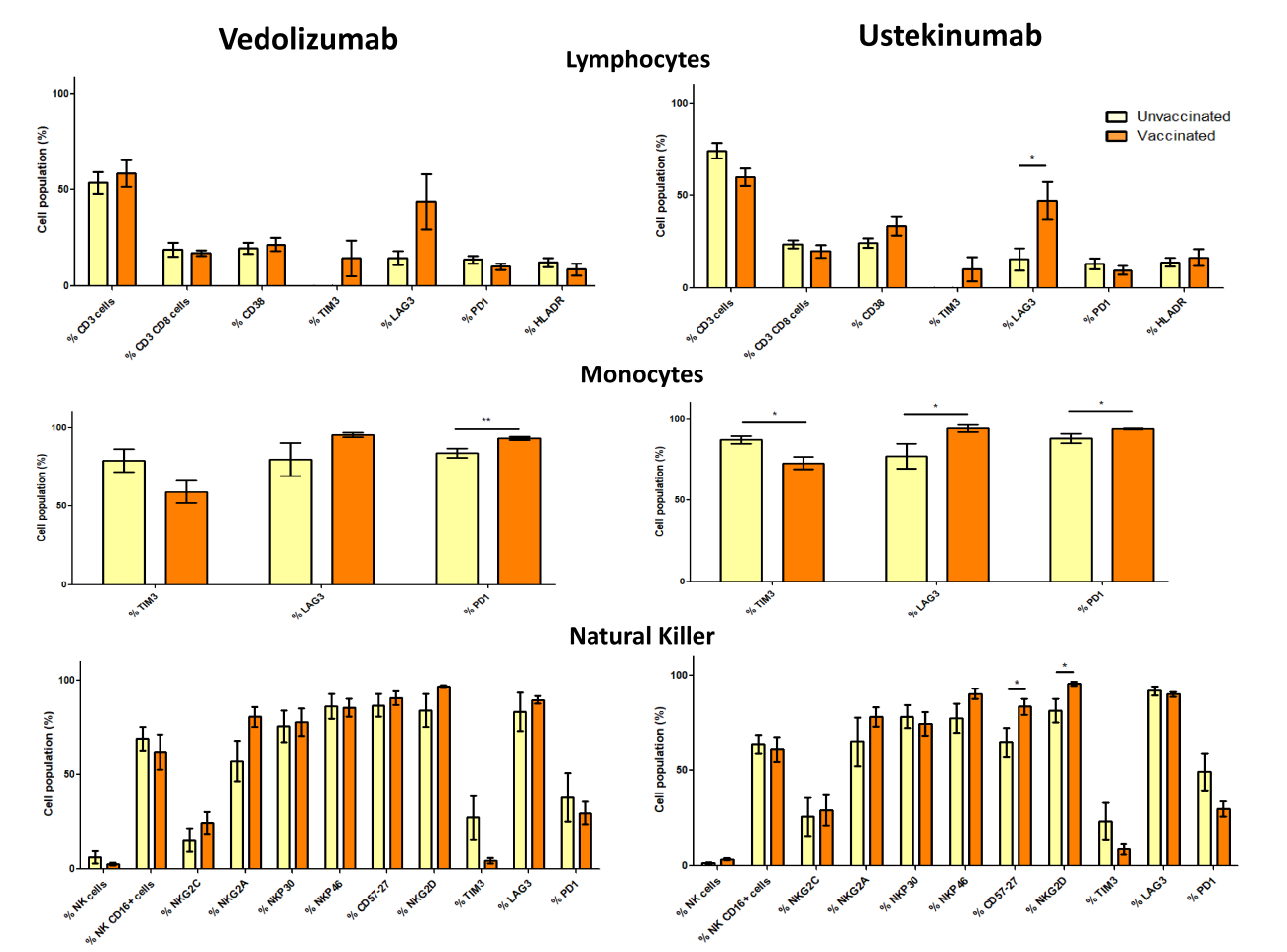
Supplementary Figure 2***. Lymphocyte, monocyte and Natural Killer populations in vaccinated and non-vaccinated patients treated with vedolizumab and ustekinumab. *p<0.05 **p<0.01 ***p< 0.001.


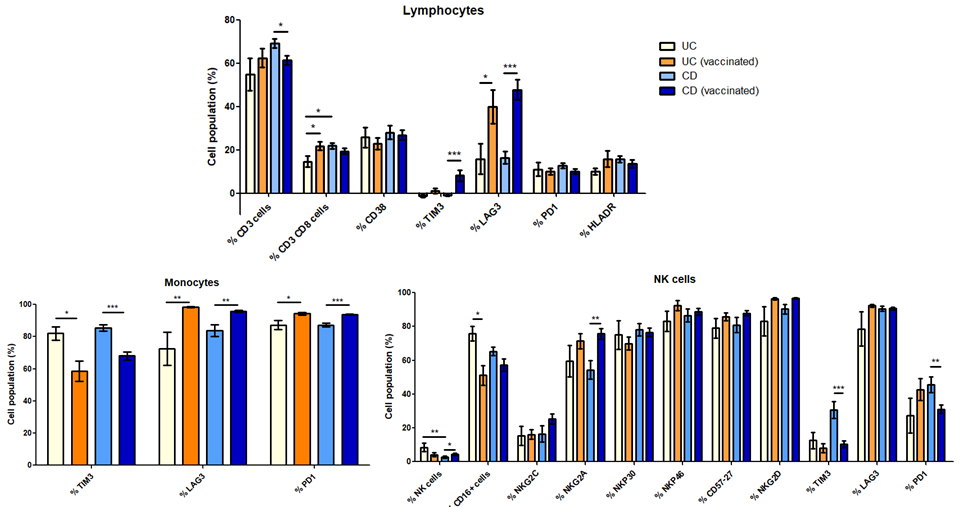


***Supplementary Figure 3***. Immune cell populations in vaccinated and non-vaccinated patients, detailed by type of IBD. CD: Crohn´s Disease. IBD: Inflammatory Bowel Disease. UC: Ulcerative Colitis.*p<0.05 **p<0.01 ***p< 0.001.


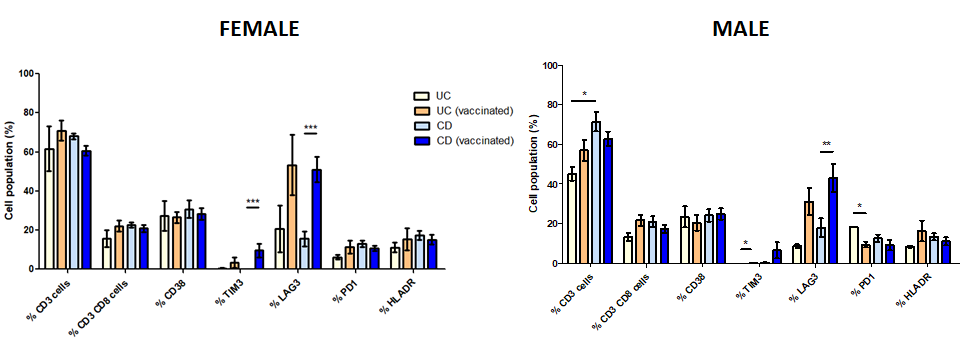


***Supplementary Figure 4***. Lymphocyte populations in vaccinated and non-vaccinated patients, detailed by gender.CD: Crohn´s Disease. UC: Ulcerative Colitis.*p<0.05 **p<0.01 ***p< 0.001.


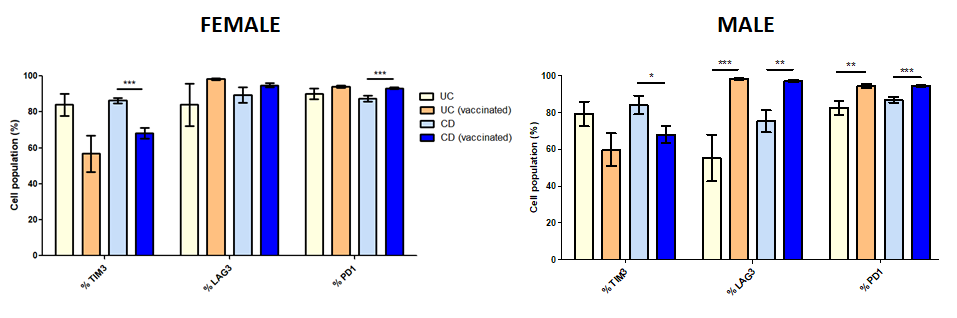


***Supplementary Figure 5***. Monocyte populations in vaccinated and non-vaccinated patients, detailed by gender.CD: Crohn´s Disease. UC: Ulcerative Colitis.*p<0.05 **p<0.01 ***p< 0.001.


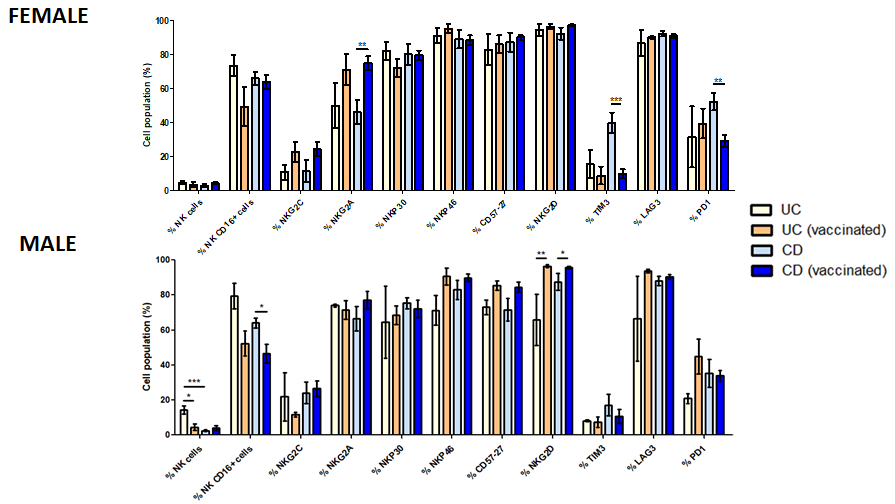


***Supplementary Figure 6***. Natural Killer (NK) populations in vaccinated and non-vaccinated patients, detailed by gender. CD: Crohn´s Disease. UC: Ulcerative Colitis. *p<0.05 **p<0.01 ***p< 0.001.


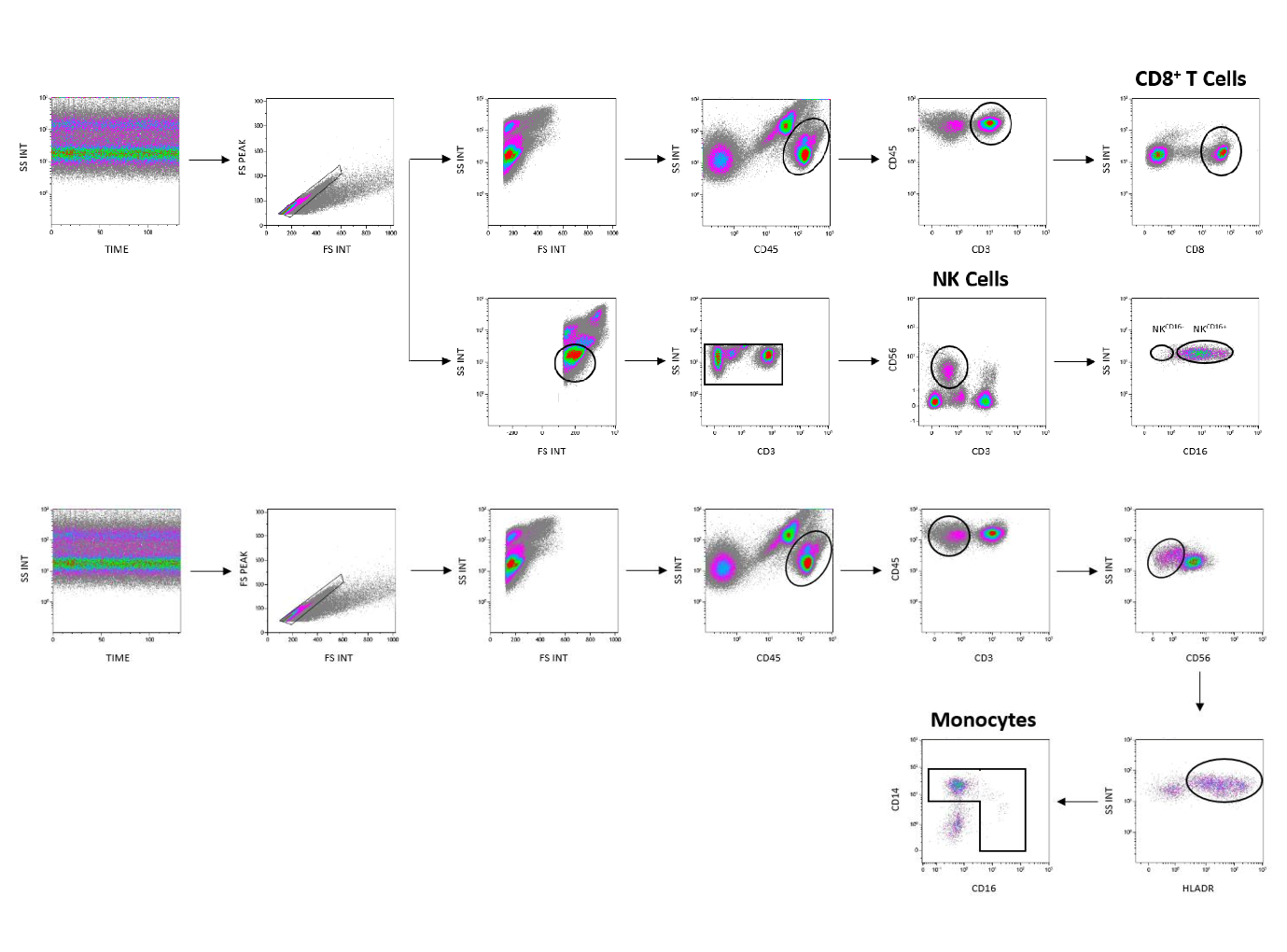


***Supplementary Figure 7***. Flow cytometry gating strategy. NK: Natural Killer. FS: Forward Scatter. SS: Side Scatter. Initially, a time gate was applied to exclude any electronic noise, followed by a singlet gate to eliminate doublets. Subsequently, total lymphocytes were gated based on a forward scatter (FS)/side scatter (SS) plot, and then further gated to isolate CD8+ T cells (CD3^+^CD8^+^), NK cells (CD3^-^ CD56^+^ CD16^+/-^), and monocyte subsets (CD3^-^ CD56^-^ HLA-DR^+^ CD14^+/-^ CD16^+/-^).
